# Time Response of Oxidative/Nitrosative Stress and Inflammation in LPS-Induced Endotoxaemia—A Comparative Study of Mice and Rats

**DOI:** 10.3390/ijms18102176

**Published:** 2017-10-18

**Authors:** Sebastian Steven, Mobin Dib, Siyer Roohani, Fatemeh Kashani, Thomas Münzel, Andreas Daiber

**Affiliations:** 1 Center for Cardiology, Cardiology I, University Medical Center of the Johannes Gutenberg-University, D-55131 Mainz, Germany; sebastiansteven@gmx.de (S.S.); mobindib@yahoo.com (M.D.); SiyerRoohani@gmx.de (S.R.); fatemehkashani_91@yahoo.com (F.K.); tmuenzel@uni-mainz.de (T.M.); 2 Center for Thrombosis and Hemostasis, University Medical Center of the Johannes Gutenberg-University, D-55131 Mainz, Germany

**Keywords:** sepsis, time response, inflammation, oxidative stress, endotoxaemia, mouse, rat

## Abstract

Sepsis is a severe and multifactorial disease with a high mortality rate. It represents a strong inflammatory response to an infection and is associated with vascular inflammation and oxidative/nitrosative stress. Here, we studied the underlying time responses in the widely used lipopolysaccharide (LPS)-induced endotoxaemia model in mice and rats. LPS (10 mg/kg; from Salmonella Typhosa) was intraperitoneally injected into mice and rats. Animals of every species were divided into five groups and sacrificed at specific points in time (0, 3, 6, 9, 12 h). White blood cells (WBC) decreased significantly in both species after 3 h and partially recovered with time, whereas platelet decrease did not recover. Oxidative burst and iNOS-derived nitrosyl-iron hemoglobin (HbNO) increased with time (maxima at 9 or 12 h). Immune cell infiltration (CD68 and F4/80 content) showed an increase with time, which was supported by increased vascular mRNA expression of *VCAM-1*, *P-selectin*, *IL-6* and *TNF-α*. We characterized the time responses of vascular inflammation and oxidative/nitrosative stress in LPS-induced endotoxaemic mice and rats. The results of this study will help to interpret and compare data from different animal species in LPS-induced endotoxaemia models for the identification of new drug targets.

## 1. Introduction

Sepsis is a clinical syndrome that is caused by a dysregulated and overshooting response of the inflammatory system to an infection. Sepsis is most frequently caused by bacteria, to a lower extend by fungi, and can rapidly become life threatening. It arises from infections of the skin, lung, abdomen, and urinary tract. Despite aggressive treatment on intensive care unit and ambitious effort in research, sepsis remains a leading cause of death, even in Western countries [1]. In 2008, costs spent on hospitalizations for sepsis in the USA were estimated at $14.6 billion [2]. The early identification of sepsis is necessary for the sufficient treatment of septic patients and scores like the modified Sepsis-related Organ Failure Assessment score (quickSOFA) are helpful tools for initiation of optimal therapy [3]. Nevertheless, the improvement of survival and reduction of costs can be best achieved by better understanding of the causes and pathophysiology of sepsis.

The basic treatment regimen for sepsis did not change for decades. Early fluid supplementation and antibiotics are known to significantly improve survival in septic shock, but clinical trials targeting the reduction of the inflammatory response failed [4]. Although there are major limitations for the translation of findings of animal studies to the human setting, it cannot be denied that animal models have significantly improved the knowledge of sepsis. In humans, sepsis is characterized by an initial pro-inflammatory phase, which is followed by an anti-inflammatory or immunosuppressive phase [5]. Although several animal models of sepsis were reported to mimic the inflammatory and genomic responses of humans to septic stimuli, this is still under debate for murine sepsis models [6,7]. An important requirement for the translation of data from animals to humans is the detailed pathophysiologic characterization of sepsis in the animal models. However, not only different protocols for the induction of sepsis, such as bacterial infusion model, cecal ligation and puncture (CLP), colon ascendens stent peritonitis (CASP), and lipopolysaccharide (LPS)-induced endotoxaemia, are used to investigate sepsis in animals. Even more problematic is the use of different species and strains in these sepsis models. In the last years, our group used LPS-injection models in mice and rats for three studies and we noticed some differences in inflammatory response and mortality between the species [8,9,10,11]. Since it is already challenging to translate findings from animal models to human sepsis, a clear characterization of the inflammatory response in each animal model of different species is highly recommended.

With the present study, we aimed to compare the time response pattern (0, 3, 6, 9, and 12 h) of white blood cell (WBC) derived oxidative and nitrosative stress, as well as vascular inflammation parameters in LPS-induced endotoxaemia in mice and rats.

## 2. Results

### 2.1. Time Response of Thrombocyte Count and White Blood Cell Derived Oxidative Burst in Mice and Rats

**Figure 1:**
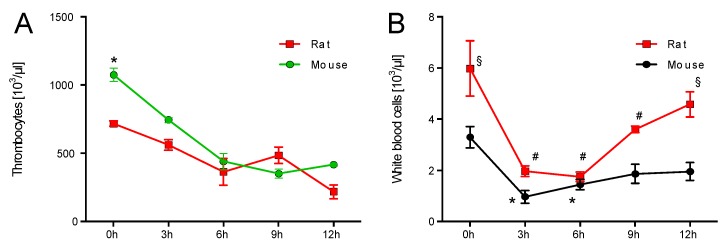

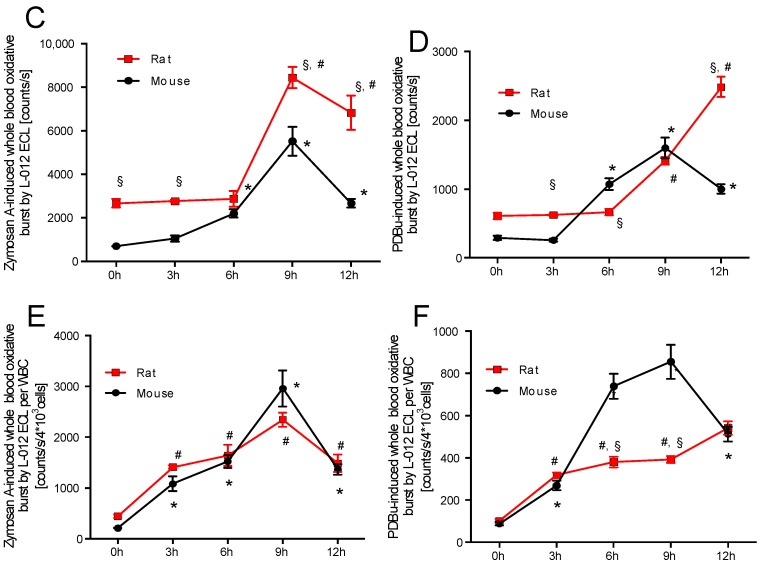
Time response of platelets and white blood cell derived oxidative burst in mice and rats. Thrombocyte count (green line already published in Steven et. al. BJP 2017 [10]) (**A**), white blood cell (WBC) count (**B**) and oxidative burst (nicotinamide adenine dinucleotide phosphate-oxidase (NADPH) oxidase and myeloperoxidase activity) in whole blood after zymosan A (**C**) or PDBu (**D**) stimulation in mice and rats was determined by chemiluminescence (L-012) over a 12 h time response. (**E**,**F**) Oxidative burst was normalized to the WBC count. The data are mean ± SEM from 6 different animals per group. * *p* < 0.05 vs. 0 h mouse, ^#^ *p* < 0.05 vs. 0 h rat and ^§^ *p* < 0.05 vs. mouse (same point in time).

Thrombocytes in whole blood were significantly higher at the beginning of the experiments. After LPS injection the thrombocyte counts in mice and rats dropped similarly (Figure 1A). Already before LPS injection, rats and mice had significantly different numbers of WBC in whole blood. In both species, the WBC count dropped significantly at 3 h after LPS injection and increased with time showing a partial normalization at 12 h. Of note, the WBC count showed better recovery in rats as compared to mice (Figure 1B). Zymosan A-induced oxidative burst in whole blood was significantly higher in rats at 0, 3, 9, and 12 h after LPS injection as compared to mice. A first significant increase of oxidative burst was detectable at 6 h after LPS injection in mice and 9 h in rats. In both species the peak level of oxidative burst was observed after 9 h (Figure 1C). A quite similar observation was made for the kinetics of PDBu-induced oxidative burst. The first significant increase with time was again detected at 6 vs. 9 h in mice as compared to rats. The maximal oxidative burst in whole blood from mice was at 9 h for zymosan A and PDBu stimulation. In contrast to the maximal zymosan A-induced oxidative burst in rats at 9 h, the peak level of oxidative burst was found at 12 h after LPS injection (Figure 1D). The absolute level of oxidative burst was higher with zymosan A than with PDBu stimulation.

Normalization of oxidative burst to WBC count in whole blood changed not only the kinetic pattern but also the relative level in the species significantly for both assays. Whereas, mice and rats showed a similar level of zymosan A-induced oxidative burst at each point in time (Figure 1E), mice showed a significantly higher PDBu-induced oxidative burst level at 6 and 9 h after LPS injection when compared to rats (Figure 1F). Finally, after 12 h burst levels were equal in both species. Of note, although the WBC count is higher in human subjects than in rodents, these numbers are comparable in rats and mice [12,13].

### 2.2. Time Response of Nitrosyl-Iron Hemoglobin and Inos Expression of Isolated WBC in Mice and Rats

**Figure 2:**
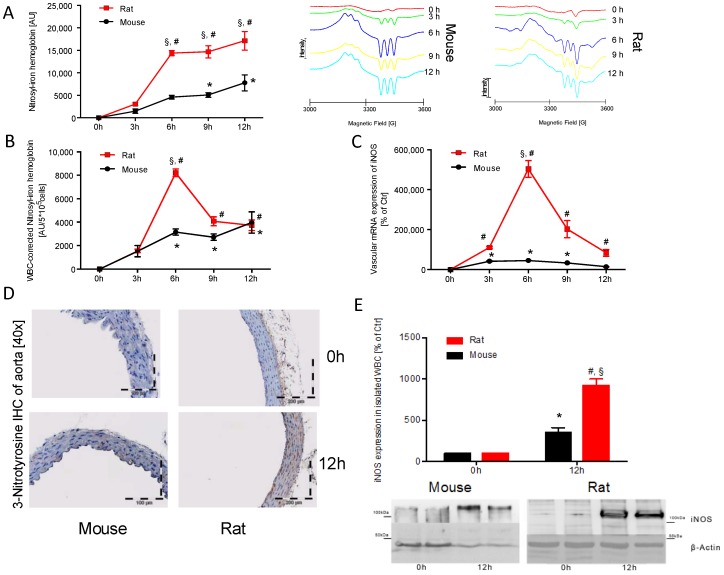
Time response of nitrosyl-iron hemoglobin and iNOS expression in isolated WBC of mice and rats. Whole blood Hb-NO levels were determined by Electron Paramagnetic Resonance (EPR) spectroscopy as a read-out of iNOS activity (**A**) and were normalized to the WBC count (**B**). qRT-PCR was used to determine mRNA expression levels of *iNOS* in aortic tissue (**C**). iNOS protein expression was further visualized by immunohistochemistry of paraffin embedded aortic sections after 12 h (**D**). iNOS protein expression was further investigated in isolated WBC of both species after 12 h using Western-blot technique (**E**). Each lane in the original blot represents a protein sample from 1–2 animals. The data are mean ± SEM from 6 different animals per group. * *p* < 0.05 vs. 0 h mouse, ^#^ *p* < 0.05 vs. 0 h rat and ^§^ *p* < 0.05 vs. mouse (same point in time).

Nitrosyl-iron hemoglobin (HbNO) significantly increased in mice after 9 h and in rats after 6 h of LPS treatment. The peak levels of HbNO were detected at 12 h after LPS injection in both species. HbNO formation was significantly (2–3-fold) higher in rats after 6, 9, and 12 h of LPS treatment as compared to mice (Figure 2A). If corrected to WBC count, the time response of HbNO levels changed. Rats demonstrated a strong peak after 6 h, whereas the time response of HbNO levels in mice showed a similar pattern as compared to the data without normalization to WBC count (Figure 2B).

Vascular mRNA of iNOS increased after 3 h of LPS treatment in both species. A substantial peak level was detected after 6 h in rats, whereas this maximum was much less pronounced in mice. mRNA expression of iNOS decreased with time and was normalized in mice, but still significantly elevated in rats at 12 h after LPS injection (Figure 2C). Immunohistochemical staining of aorta revealed an increase in 3-nitrotyrosine positive proteins throughout the entire vascular wall in both species after 12 h, although the staining was more pronounced in rats as compared to mice (Figure 2D). iNOS expression in isolated WBC of mice and rats was elevated at 12 h after induction of endotoxaemia (Figure 2E). However, rats showed a significantly higher iNOS protein expression as compared to mice.

### 2.3. Time Response of Vascular Inflammation in Mice and Rats

**Figure 3:**
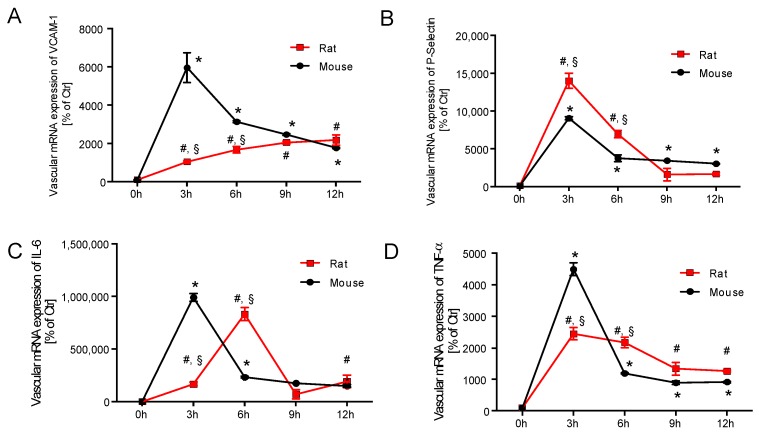
Time response of vascular inflammation in mice and rats. qRT-PCR was used to determine mRNA expression levels of *VCAM-1* (**A**), *P-selectin* (**B**), *IL-6* (**C**) and *TNF-α* (**D**) in aortic tissue over a 12 h time response. The data are mean ± SEM from 6 different animals per group. * *p* < 0.05 vs. 0 h mouse, ^#^ *p* < 0.05 vs. 0 h rat and ^§^ *p* < 0.05 vs. mouse (same point in time).

*VCAM-1* mRNA expression increased significantly with time in endotoxaemic mice and rats. *VCAM-1* expression in mice showed a maximum at 3 h after LPS injection (3-fold higher compared to rats) and *VCAM-1* expression declined after the 3 h maximum but was still significantly higher at all points in time compared to 0 h. The maximum *VCAM-1* expression in rats was found after 12 h of LPS treatment with a similar amount as compared to mice and expression levels continuously increased with time in rats (Figure 3A). Vascular mRNA expression of *P-selectin* showed a peak after 3 h of LPS treatment in both species and time-dependently returned to normal levels afterwards. The absolute expression levels and time responses were almost identical for both species (Figure 3B). *IL-6* mRNA expression showed a maximum at 3 h after LPS injection in mice and at 6 h in rats. Afterwards, the *IL-6* levels were normalized in both species with an overall almost identical time response (Figure 3C). *TNF-α* mRNA expression showed a peak at 3 h after LPS injection in both species, but this maximum was less pronounced in rats as compared to mice. Expression levels were higher at all time response compared to 0 h values in mice and rats (Figure 3D).

**Figure 4:**
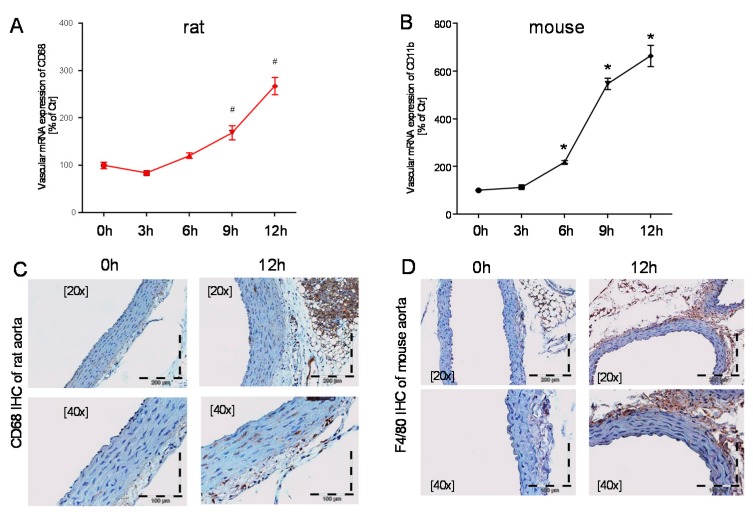
Time response of vascular inflammation in mice and rats. qRT-PCR was used to determine mRNA expression levels of *CD68* (**A**) in rats and *CD11b* in mice (**B**). CD68 protein levels in rats (**C**) and F4/80 protein levels in mice (**D**) were further visualized by immunohistochemistry of paraffin embedded aortic sections after 12 h. The data are mean ± SEM from 6 different animals per group. * *p* < 0.05 vs. 0 h mouse, ^#^ *p* < 0.05 vs. 0 h rat (same point in time).

mRNA expression of *CD68*, as a marker for leukocyte infiltration, was increased at the 9 and 12 h point in time (Figure 4A). Increased CD68 positive protein was also detected by immunohistochemical staining of rat aorta at 12 h after LPS injection (Figure 4C). *CD11b* mRNA levels in vascular tissue showed a time-dependent significant increase in mice at the 6, 9, and 12 h points in time (Figure 4B). F4/80 immunohistochemical staining of mouse aorta revealed more F4/80 positive protein throughout the entire vascular wall after 12 h of LPS treatment (Figure 4D). Whereas, CD68 and F4/80 are good markers for infiltrated macrophages, CD11b is a rather unspecific marker that is expressed on all myelomonocytic cells.

## 3. Discussion

In this study, we provide very detailed time responses (0, 3, 6, 9, and 12 h) of oxidative/nitrosative stress and vascular inflammation in LPS-induced endotoxaemia in mice and rats. We also provide a detailed comparison of these time responses between mice and rats to identify species-independent pathways that might be of relevance for human sepsis as well. LPS-induced endotoxaemia is a frequently used model for preclinical research on sepsis. However, so far, all published studies used different points in time and multiple species, which makes it hard to compare the disease-relevant pathomechanisms and translate the findings to the human setting. Besides species-dependent differences in the inflammatory response, there are even notable differences between strains of the same species. It is known, that C57BL/6j (B6) tend to have a TH1-predominat response to pathogens, whereas A/J, BALB/C, and DBA/2 mice have a TH2-type preference [14]. Furthermore, B6 mice when compared to A/J mice have a significant higher mortality, which was accompanied by higher IL-10 plasma levels and myeloperoxidase activity [15]. The present study underlines the importance of using similar time points, strains and species, and highlights some alarming differences in the time responses and absolute changes across two animal species warranting cautious interpretation of data from experimental studies when translating them to human sepsis.

The endotoxin LPS from gram-negative bacteria triggers inflammation by binding directly via CD14 on monocytes and via toll-like receptors (TLR) on endothelial cells after forming a complex with LPS binding protein (LBP) [16]. The LPS model nicely mirrors the severe inflammatory response of the organism but does not challenge the host with living bacteria. Anti-inflammatory treatment of sepsis should counteract the excessive inflammatory cascade and contribute to improved survival. Statins have such anti-inflammatory properties and a recent trial tested the use of rosuvastatin in patients suffering from acute respiratory distress syndrome (ARDS) but the results were disappointing and statin therapy failed to improve clinical outcome [17]. Several other compounds were tested in clinical and pre-clinical trials to find evidence for improved survival by the reduction of the inflammatory response in sepsis, but only cortisone therapy can be found in recent clinical guidelines [4].

The importance of time responses for our understanding of the septic pathophysiology is underlined by literature reports on different time windows or phases for human sepsis but also LPS-induced endotoxaemia [8,18]: the first phase is between 0 and 2 h after bacterial invasion/LPS exposure, and the second phase between 2 and 12 h. Within these two phases, pathophysiological processes (cell activation, inflammation, and hypotension but also local endothelin-1 formation [8,18,19]) are reversible and can be pharmacologically modified [18]. The upregulation of cell activation markers and inflammation were also observed in the present study (e.g., CD68, F4/80, VCAM-1). After these two phases, a point of no return was postulated, and all later events contribute directly to the high mortality (e.g., massive oxidative damage, cell death, disseminated intravasal coagulation (DIC), and end organ failure), and cannot be easily modified by pharmacotherapy [8]. According to a clinical study of Vargas et al. the decision on survival or death in septic shock develops between 40 and 60 h post infection (probably the decision is already taken somewhat earlier, between 12 and 24 h) and can be predicted from the serum pattern of the inflammation markers IL-6 and IL-8 as well as the adhesion molecules sELAM-1 and sICAM-1 [20]. After 40 h, the level of these inflammation markers returned to normal in the survivor group but escalated in the non-survivor group. Another clinical study showed that the serum levels of the regulator of endothelial cell migration and endothelial permeability VEGF-A as well as its receptor sFlt-1 were significantly upregulated (48 h after the onset of fever) in patients with septic shock but were at normal level in patients who had developed sepsis without shock [21]. Therefore, according to this study, the decision on development of shock or “mild” sepsis was already taken at 48 h post infection. In an animal experimental study on septic sheep, Lange et al. presented data on the time response of different clinical and biochemical markers and revealed a dramatic change in the expression of NOS isoforms between 8 and 12 h post infection, which was followed by severe worsening of the clinical parameters [22].

In addition, a large number of studies investigated the time response of one marker in plasma or serum such as angiopoietin-2, gelsolin, neopterin, C-reactive protein (CRP), and selenium. In summary, despite many efforts it is not clear how the septic situation in a given animal develops to the critical point at which the vascular biochemistry and physiology either return to normal or develop into the lethal situation of septic shock. It is also unclear which animal model should be applied to mimick human sepsis but some data from patients are helpful in pointing out the crucial steps involved. In the first place this applies to the high output of prostacyclin (PGI_2_, measured as 6-keto-PGF_1α_) before shock develops [23,24,25,26]. Of clinical importance is the fact that high levels of 6-keto-PGF_1α_ are associated with a bad prognosis of septic patients [23]. One may speculate that similar to the inhibition of PGI_2_ synthesis in the endothelium after 1 h of LPS exposure the beneficial effects of smooth muscle derived PGI_2_ after 4–12 h could be eliminated by the inhibition of its synthesis, which could either be brought about by inhibition of inducible cyclooxygenase (COX-2) (e.g., downregulation or nitration of COX-2 [27]) or even by nitration of PGI_2_ synthase after breakdown of the smooth muscle antioxidant potential, followed by a rise of the peroxynitrite levels in this previously resistant cell layer. Of note, despite the presence of severe hypotension, a considerable degree of vascular dysfunction may exist, a paradox that is best explained as follows: the substantial stimulation of the sGC/cGMP signaling cascade with high formation rates of ^•^NO from iNOS (as shown here by EPR-based measurement of nitrosyl-iron-Hb) may result in a desensitization of this signaling pathway, endothelial dysfunction, and vasoconstriction [28,29]. This would provide a reasonable explanation for the impaired vascular function despite systemic hypotension and overproduction of vasodilators. Therefore, the time window between 12 and 24 h may have special significance for the development of lethality.

In the third phase, NOS-2 and COX-2 expression are decreased, PGI_2_ synthase is nitrated/inactivated due to huge amounts of ROS and peroxynitrite from infiltrated leukocytes and endothelial dysfunction, at least in the larger vessels, is observed. At this time point (upon loss of vasodilators), enhanced thrombosis is encountered [30], as also observed in our LPS treated mice [10]. The relevance of microthrombi formation (disseminated intravascular coagulation) for the prognosis of septic patients was also demonstrated by an increased mortality of septic patients with reduced numbers of circulating platelets (thrombocytopenia) [31], as also observed in our LPS treated rats and mice [10,11]. This represents another hint for the loss of the potent antiaggregatory compounds PGI_2_ and ^•^NO. Therefore, the impaired vascular function observed in our previous studies, in vessels from LPS-treated rats and mice, is in good accordance with the clinical and experimental time response of sepsis [8,10,11]. For LPS-treated rats previous studies have shown substantial hypotension at 6 h after LPS treatment and increase in mean arterial pressure afterwards [19,32]. This would be in good accordance with the here postulated third phase of sepsis, characterized by impaired vascular relaxation and even a mild hypertension (at least restricted to certain vessel areas or organs) as well as the previously reported increased endothelin-1 levels can be observed in the late phase of sepsis.

Previous studies from our group and others show that median survival of C57/Bl6j mice upon i.p. injection of 10 mg/kg LPS is approximately 20 h after injection, whereas the median survival rate of Wistar rats challenged with the same amount of LPS intra peritoneal was shorter (6–10 h) [8,11,33]. Interestingly, mRNA levels of inflammatory makers like *VCAM-1, IL-6,* and *TNF-α* were significantly higher and had an earlier peak (*IL-6* and *VCAM-1*) in mice as compared to rats, which reflects a stronger and earlier inflammatory response in mice compared to rats. However, the time response and absolute level of nitrosative/oxidative stress rather showed an opposite pattern. In both species WBC count in whole blood decreased significantly after 3 h of LPS treatment, but only in rats the number of WBC recovered significantly. Interestingly, the absolute WBC-derived oxidative burst was significantly higher in rats when compared to mice at 0, 3, 9, and 12 h points in time.

NO mediates several beneficial effects like vasodilation, inhibition of platelet aggregation, anti-inflammatory and anti-apoptotic effects via activating soluble guanylate cyclase (sGC) [34]. In the setting of septic shock excessive generation of ^•^NO by the iNOS in combination with excessive production of superoxide (O_2_^•^^−^) by NADPH oxidase of WBC leads to generation of peroxynitrite (ONOO^−^). The latter is capable to affect enzyme function by oxidation or nitration, induces cell damage by lipid peroxidation, and attacks mitochondrial enzymes leading to mitochondrial dysfunction [35]. Our data demonstrate in detail, that ^•^NO generation in total is significantly higher in rats 6 h after induction of sepsis, which was due to increased expression of iNOS in WBC and vascular tissue. Together with the high burden of superoxide generation a strong peroxynitrite footprint was detectable by immunohistochemical staining of vascular tissue. Taken together, the burden of peroxynitrite formation in rats was higher than in mice.

## 4. Materials and Methods

### 4.1. Materials

The Bradford reagent was obtained from BioRad (Munich, Germany). The QuantiTect probe RT-PCR Kit was purchased from Qiagen (Hilden, Germany). All TaqMan probes were purchased from Applied Biosystems (Darmstadt, Germany). L-012 (8-amino-5-chloro-7-phenylpyrido[3,4-d] pyridazine-1,4-(2*H*,3*H*)dione sodium salt) was purchased from Wako Pure Chemical Industries (Osaka, Japan). For the induction of septic shock, we used LPS from *Salmonella Thyphosa* from Sigma-Aldrich (St. Louis, MO, USA). All of the other chemicals were of analytical grade and were obtained from Sigma-Aldrich, Fluka (St. Louis, MO, USA) or Merck (Darmstadt, Germany).

### 4.2. Animals and In Vivo Treatment

All of the animal care and experimental procedures were in accordance with the Guide for the Care and Use of Laboratory Animals as adopted by the US National Institutes of Health, and approved by the Ethics Committee of the University Medical Center Mainz and the Landesuntersuchungsamt Koblenz (#23 177-07/G 14-1-039, 12 June 2014). In this study, a total number of 30 C57BL/6j mice and 30 Wistar rats received single injection of LPS intraperitoneally at a dosage of 10 mg/kg for both species. Animals of both species were divided into five groups, six animals each, and sacrificed by exsanguination under isoflurane anesthesia (5% inhalant in room air) at 0, 3, 6, 9, and 12 h after injection of LPS. All C57BL/6j mice were male, and weighed 25–30 g at the time of the experiment. All of the Wistar rats were male, and weighed 270–330 g at the time of the experiment.

### 4.3. Chemiluminescence-Based Detection of Oxidative Stress in Whole Blood

Whole blood oxidative burst mainly reflects leukocyte NADPH oxidase and myeloperoxidase activity, and was used as a global readout of the burden of oxidant formation as well as of the activation state of inflammatory pathways. To measure whole blood leukocyte-dependent ROS formation, fresh blood (in citrate tubes) was stimulated with phorbol ester PDBu (10 µM) or the fungal endotoxin zymosan A (50 µg/mL) and assessed in PBS containing Ca^2+^/Mg^2+^ (1 mM) by L-012 (100 µM) enhanced chemiluminescence (ECL) using a Centro chemiluminescence plate reader from Berthold Technologies (Bad Wildbad, Germany) [8].

### 4.4. Quantification of Nitrosyl-Iron Hemoglobin in Whole Blood by Electron Paramagnetic Resonance (EPR) Spectroscopy

NO synthesis and the burden of nitrosative stress were also assessed by the EPR-based detection of Hb-NO. Samples of venous blood for Hb-NO/EPR studies were obtained by the puncture of the right heart of anesthetized mice and rats; blood samples were immediately frozen and stored in liquid nitrogen. The EPR measurements were carried out at 77 K using an X-band table-top spectrometer MS400 from Magnettech (Berlin, Germany). The instrument settings were as follows: 10 mW microwave power, 7000 mG amplitude modulation, 100 kHz modulation frequency, 3300 G center field, 300 G sweep width, 60 s sweep time and three scans, as described before [11].

### 4.5. Isolation of White Blood Cells

The procedure was described before [36]. Briefly, erythrocytes in mice and rat blood were separated by sedimentation in 15 mL heparin-supplemented tubes after addition of an equal volume of dextran solution (MW 485,000, T500 9219.1 from Roth). 1.9 g dextran were dissolved in 50 mL 0.9% NaCl solution. The leukocyte-containing supernatant was centrifuged on Histopaque-1083 from Sigma Aldrich for 30 min at 500× *g* at 20 °C, resulting in a neutrophil (PMN)-containing pellet and the monocyte/lymphocyte-enriched (WBC) “buffy coat” between the aqueous and Ficoll phases. The WBC fraction was collected and purified by further centrifugation for 10 min at 500× *g,* followed by resuspension in PBS. The PMN pellet was freed from residual erythrocytes by hypotonic lysis in distilled water and centrifugation at 500× *g* (two to three times). The total blood cell count and the purity of the fractions were evaluated using an automated approach using a hematology analyzer KX-21N from Sysmex Europe GmbH (Norderstedt, Germany). Typical content of WBC in each fraction was previously published [37].

### 4.6. Western Blot Analysis

Isolated white blood cell proteins from mice and rats were separated by 7.5% SDS-PAGE under reducing conditions. After blotting on a nitrocellulose membrane, immunoblotting was accomplished using antibodies against β-actin (rabbit, monoclonal, 1:2500, Sigma-Aldrich, Seelze, Germany), and iNOS (purified anti iNOS, mouse, monoclonal, 1:2500, BD Biosciences, Franklin Lakes, NJ). Detection was accomplished with either Super Signal Substrate (Pierce, Rockford, IL, USA) or ECL Reagent (Amersham, Piscataway, NJ, USA). Bands were evaluated by densitometry.

### 4.7. Reverse Transcription Real-Time PCR (qRT-PCR)

mRNA expression was analyzed with quantitative real-time RT-PCR, as previously described [38]. RNA from aorta and white blood cells was isolated in both species for the experiments. Briefly, total RNA from mouse and rat aorta, and from isolated whole blood cells of mice and rats was isolated (RNeasy Fibrous Tissue Mini Kit; Qiagen, Hilden, Germany), and 50 ng RNA was used for real-time RT-PCR analysis with the QuantiTect Probe RT-PCR kit (Qiagen). TaqMan^®^ gene expression assays for *vascular cell adhesion molecule 1* (*VCAM-1*), the cytokine *interleukin 6* (*IL-6*), the adhesion molecule *P-selectin*, the immune-signaling protein *tumor necrosis factor-α* (*TNF-α*), as well as the house keeping gene *TATA-box binding protein* (*TBP*) were purchased as probe-and-primer sets (Applied Biosystems, Foster City, CA) for mice and rats. TaqMan^®^ gene expression assays for leukocyte marker *CD68* in mice and *macrophage-1 antigen* also known as *CD11b* in rats from the same provider were also tested. The comparative Delta Ct method was used for relative mRNA quantification. Gene expression was normalized to the endogenous control, *TBP* mRNA, and the amount of target gene mRNA expression in each sample was expressed relative to that of control for every species.

### 4.8. Immunohistochemistry and Fluorescence Microscopy

For immunohistochemistry aorta segments from rats and mice were fixed in paraformaldehyde (4%) and embedded in paraffin. Aortic segments from both species were stained with primary antibodies against 3-nitrotyrosine (Millipore, Burlington MA, USA). Mouse aortic segments were also stained with primary antibodies against F4/80 (eBioscience) and rat aortic segments were also stained for CD68 (LS Bio, Seattle, WA, USA). Anti-rat, anti-rabbit, and LSAB (Vector; Sigma; DAKO, Glostrup, Denmark, respectively) were used as secondary antibodies. For immunohistochemical detection ABC reagent (Vector, Burlingame, CA, USA) and then DAB reagent (peroxidase substrate Kit, Vector, Burlingame, CA, USA) were used.

### 4.9. Statistical Analysis

Results are expressed as mean ± SEM. Two-way ANOVA (with Holm-Sidak’s correction for comparison of multiple means) was used for comparisons of time responses of WBC, mRNA expression (*VCAM-1*, *P-selectin*, *IL-6*, *TNF-α*), HbNO-levels and oxidative burst. One-way ANOVA (with Bonferroni’s correction for comparison of multiple means) was used for comparisons of mRNA expression (*CD11b*, *CD68*) and iNOS protein levels. *p* values < 0.05 were considered as statistically significant. All of the statistical analyses were performed using GraphPad Prism 6.0d.

## 5. Conclusions

Human sepsis is poorly understood and more research is needed to understand the pathomechanisms. Sepsis studies are often performed in different species (mice and rats) and even different strains (C57Bl6/j, BALB/c, A/J) challenged with LPS or CLP-procedure and the results are compared to each other. Nevertheless, the models are poorly characterized, especially with respect to time responses for the entire set of parameters. With the present study, we present species specific, time-dependent changes of regulators of cell activation, inflammation, and oxidative/nitrosative stress in mice and rats after LPS-challenge. Based on our present findings, the survival of endotoxaemic animals might rather be related to suppression of oxidative/nitrosative stress than to complete suppression of inflammatory responses. These data can help to interpret sepsis studies performed in models of LPS-induced endotoxaemia in mice and rats and translate these data to the human setting. Based on this study, further time- and species-dependent characterization of sepsis relevant parameters like hemostasis, vascular function, or immune cell differentiation and recruitment should be conducted.

## Abbreviations

LPS	Lipopolysaccharide
WBC	White blood cell
HbNO	nitrosyl-haemoglobin
iNOS	inducible NOS (type 2)
IL-6	Interleukin-6
TNF-α	tumor necrosis factor-α
VCAM-1	vascular adhesion molecule-1
PDBu	Phorbol 12,13-dibutyrate
